# A Convenient Method for the Synthesis of (Prop-2-Ynyloxy)Benzene Derivatives via Reaction with Propargyl Bromide, Their Optimization, Scope and Biological Evaluation

**DOI:** 10.1371/journal.pone.0115457

**Published:** 2014-12-29

**Authors:** Tannaza Batool, Nasir Rasool, Yasmeen Gull, Mnaza Noreen, Faiz-ul-Hassan Nasim, Asma Yaqoob, Muhammad Zubair, Usman Ali Rana, Salah Ud-Din Khan, M. Zia-Ul-Haq, Hawa Z. E. Jaafar

**Affiliations:** 1 Department of Chemistry, Government College University Faisalabad, Faisalabad, Pakistan; 2 Department of Chemistry, Islamia University of Bahawalpur, Bahawalpur, Pakistan; 3 Deanship of scientific research, College of Engineering, King Saud University, Riyadh, Saudi Arabia; 4 The Patent Office, Karachi, Pakistan; 5 Department of Crop Science, Faculty of Agriculture, 43400 UPM Serdang, Selangor, Malaysia; University of East Anglia, United Kingdom

## Abstract

A highly convenient method has been developed for the synthesis of (prop-2-ynyloxy) benzene and its derivatives. Differently substituted phenol and aniline derivatives were allowed to react with propargyl bromide in the presence of K_2_CO_3_ base and acetone as solvent. The compounds were synthesized in good yields (53–85%). Low cost, high yields and easy availability of compounds helped in the synthesis. Electron withdrawing groups favor the formation of stable phenoxide ion thus in turn favors the formation of product while electron donating groups do not favor the reaction. Phenol derivatives gave good yields as compared to that of aniline. As aprotic polar solvents favor S_N_2 type reactions so acetone provided best solvation for the reactions. K_2_CO_3_ was proved to be good for the synthesis. Antibacterial, Antiurease and NO scavenging activity of synthesized compounds were also examined. 4-bromo-2-chloro-1-(prop-2-ynyloxy)benzene **2a** was found most active compound against urease enzyme with a percentage inhibition of 82.00±0.09 at 100 µg/mL with IC_50_ value of 60.2. 2-bromo-4-methyl-1-(prop-2-ynyloxy)benzene **2d** was found potent antibacterial against *Bacillus subtillus* showing excellent inhibitory action with percentage inhibition of 55.67±0.26 at 100 µg/ml wih IC_50_ value of 79.9. Based on results, it can be concluded that some of the synthesized compounds may have potential antiurease and antibacterial effects against several harmful substances.

## Introduction

(Prop-2-ynyloxy)benzene is an important compound having vast synthetic applications. Most important of all of its applications is its use as triazole intermediate [1]. Synthetic methods of organic chemistry reveal alkyne groups to be most reactive ones which increase the importance of the compound [2]. Low cost, high yield of the compound has attracted the attention of scientists to synthesize the compound as monomer for many polymeric compounds as it is thermally reactive end capping agent. [3] The reaction became much more important due to presence of terminal alkyne which helps in easy polymerization reactions under mild reaction condition, low temperature which is more favorable for linear and graft polymeric compounds. [4] (Prop-2-ynyloxy)benzene structure is mainly found in compounds of medicinal importance specially of antimicrobial importance. [5] (Prop-2-ynyloxy)benzene type compounds are also used in synthesis of large number of polymeric compounds mainly included lignin [6], azide functionalized dendrons [7], and β lactam fused enediynes [8], mono and bis-1,2,3-triazole acyclonucleoside [9], symmetrical and asymmetrical, cyclic and acylic enediynes [10] and sulfonated polytriazole proton exchange membrane for methanol feul cells via click chemistry. [4] Synthetic importance of the compound is revealed from its structure having easy approach for upcoming substances joining them to form complex molecules more accurately with great ease [11].

Haung and coworkers observed that (Prop-2-ynyloxy)benzene type compounds showed antimicrobial, antitumor, anti HIV, DNA scavenging inhibitors, anti proliferating, anti obesity and central nervous system activities. [4] The main intention of the present work is the synthesis of (Prop-2-ynyloxy)benzene derivatives by reported methodologies [12]–[20] and our focused to adopt optimization reaction condition such as effect of solvents, moler concentration of bases, stirring and refluxing time. In addition evaluating their antibacterial, antiurease and NO scavenging activities.

## Results and Discussion

### Chemistry

Krim and co-workers reported that (Prop-2-ynyloxy)benzene is an intermediate in the synthesis of triazole via Huigen dipolar cycloaddition method using click chemistry. Terminal alkynes provide an efficient method for the synthesis of triazole, [21] which can be prepared by reaction of 3-Bromopropyne and aniline derivatives in DMF [9], [22]. We reported the reaction of phenol and propargyl bromide for the synthesis of (Prop-2-ynyloxy)benzene derivatives under different reaction conditions. [23]–[28] There is very little work on the synthesis of reported compounds under these optimized reaction conditions and so far no information was published for antibacterial, antiurease and NO scavenging activity of the synthesized compounds.

**Figure 1:**
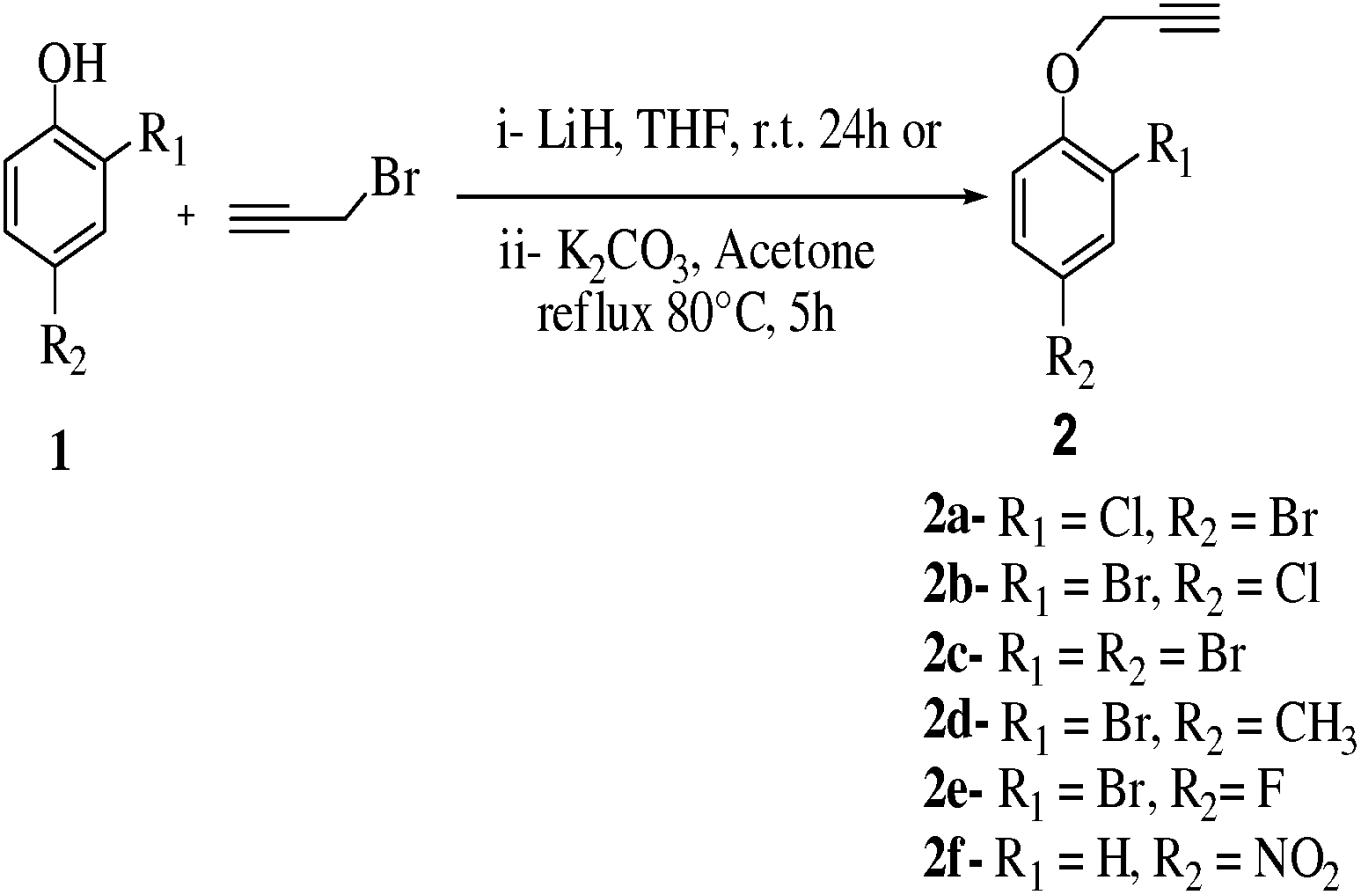
Synthesis of compounds 2a–f.

We optimized the reaction conditions like temperature, solvents, bases and its concentration for the synthesis of compounds **(2a–2f)** (Fig. 1, Table 1)**.** It was observed that successive addition of various bases and solvent types helped to obtain moderate to very good yields (74–85%) of synthesized compounds. For the synthesis of compound **(2d)** 2-bromo-4-methyl-1-(prop-2-ynyloxy) benzene, 2-bromo-4-methyl phenol (0.535 mmol) was reacted with propargyl bromide (1.2 eq.) in THF using LiH (2 eq.) as base at room temperature producing no product. It was noted that **2d** was synthesized with almost 74% yield, when the reaction was carried out in acetone and K_2_CO_3_ (2 eq.) under reflux conditions for 5 hrs. Compound, 1-nitro-4-(prop-2-ynyloxy) benzene **(2f)** was prepared by using dry acetone and K_2_CO_3_ (3.5 eq.) under reflux conditions for 5 hour producing 76% yield. However when compound was first stirred in the presence of K_2_CO_3_ (3.5 eq.) and acetone at room temperature for 2 hour then adding propargyl bromide (1.3 eq.) to the reaction mixture and stirring for 16 hour resulted in 53% yield. It was surprisingly noted that acidity of phenoxide ion was also affected by nature of substitutions. Electron withdrawing groups might increase the acidity to led enhanced yield of products while electron donating groups allowed to decrease the yield of synthesized compounds (Fig. 1, Table 1).

**Table 1:** Optimization of reaction conditions for compounds **2a–2f**.

Entry	Reactant	Product	Solvents	Base	Temp.	Time*	Yield*
1	C_6_H_4_OBrCl	C_9_H_6_OClBr **2a**	Acetone	K_2_CO_3_	80°C	5 h	79%
2	C_6_H_4_OBrCl	C_9_H_6_OClBr **2b**	Acetone	K_2_CO_3_	80°C	5 h	78%
3	C_6_H_4_OBr_2_	C_9_H_6_OBr_2_ **2c**	Acetone	K_2_CO_3_	80°C	5 h	85%
4	C_9_H_7_OBr	C_10_H_9_OBr **2d**	THF	LiH	r.t	24 h	0%
			Acetone	K_2_CO_3_	80°C	5 h	74%
5	C_6_H_4_OBrF	C_9_H_6_OBrF **2e**	Acetone	K_2_CO_3_	80°C	5 h	79%
6	C_9_H_5_O_3_N	C_9_H_7_O_3_N **2f**	Acetone	K_2_CO_3_	80°C	30 h	53%
			Acetone	K_2_CO_3_	80°C	5 h	76%

*Time checked by TLC.

*Isolated Yield.

**Figure 2:**
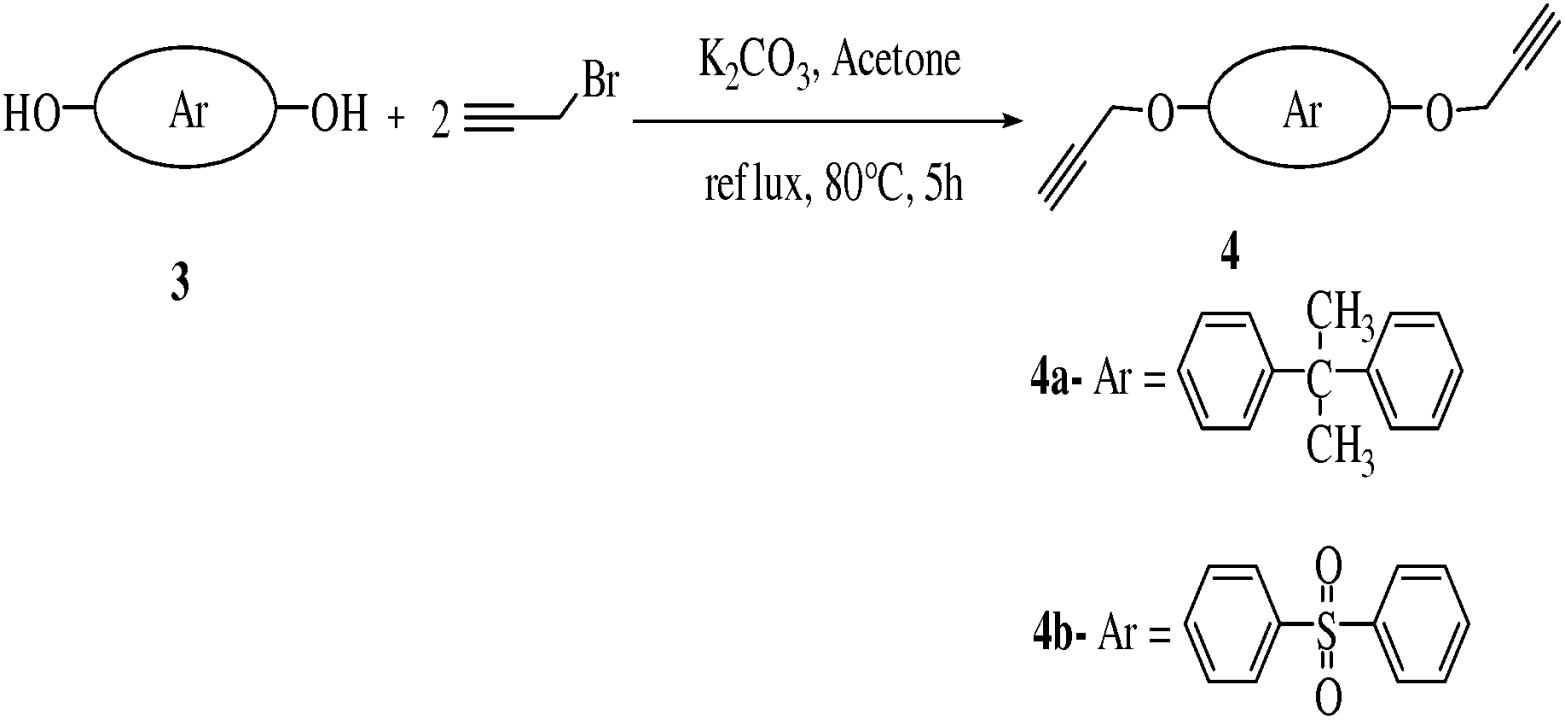
Synthesis of compounds 4a–b.

Compound **4a** and **4b** were prepared by reaction of 4,4′-(propane-2,2-diyl)diphenol (0.87 mmol) and 4,4′-sulfonyldiphenol (0.8 mmol) with propargyl bromide (2.4 eq.) in acetone using K_2_CO_3_ (4 eq.) as base under reflux conditions (80°C) respectively. (Fig. 2, Table 2). It was noteworthy that **4b** gave very good yield (73%) due to more acidic protons of 4,4′-sulfonylbis((prop-2-ynyloxy)benzene) while **4a** gave moderate yield (70%) of 4,4′-(propane-2,2-diyl)bis((prop-2-ynyloxy)benzene) due to less acidic protons because of two methyl moieties present in the molecule.

**Table 2:** Optimization of reaction conditions for compounds **4a–b**.

Entry	Reactant	Product	Base	Solvent	Time	Temp.	Yield
**1**	C_15_H_16_O_2_	C_21_H_20_O **4a**	K_2_CO_3_	Acetone	5 h	80°C	70%
**2**	C_12_H_10_O_4_S_2_	C_14_H_14_S_2_O_2_ **4b**	K_2_CO_3_	Acetone	5 h	80°C	73%

**Figure 3:**
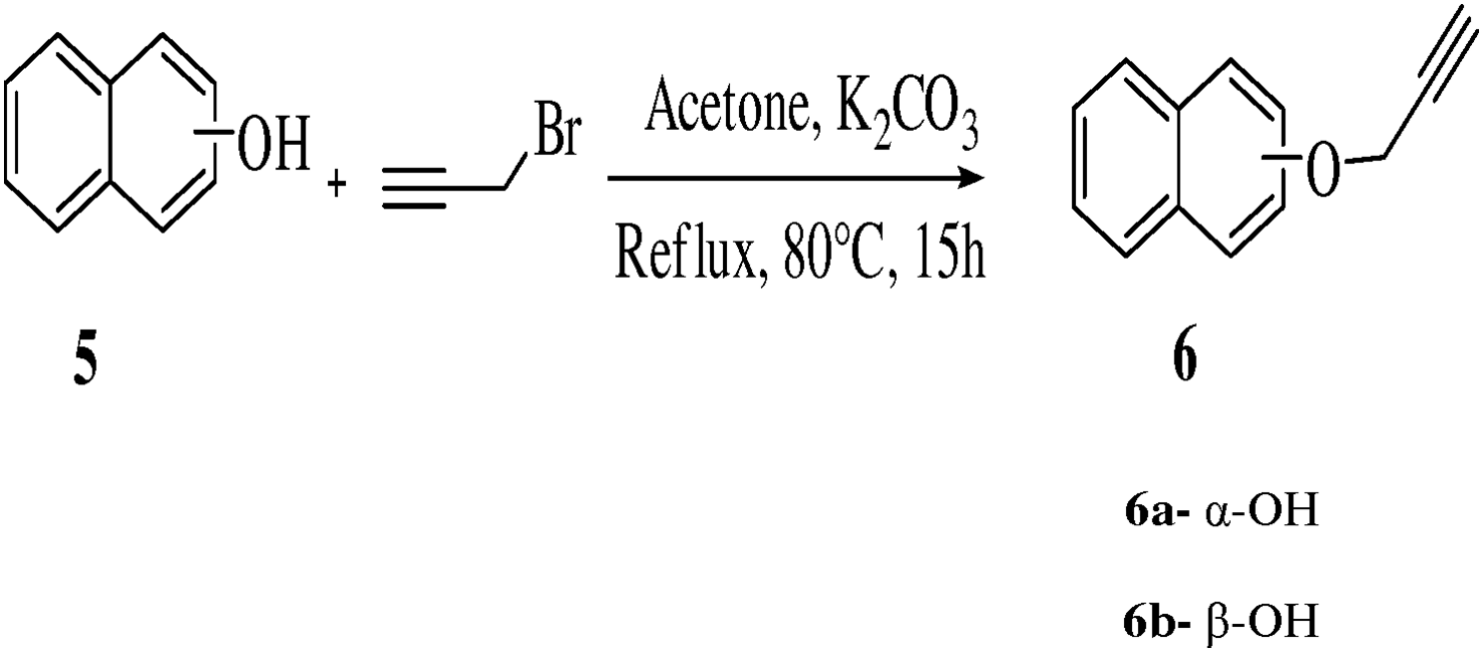
Synthesis of compounds 6a–b.

It was observed that naphthalene-1-ol (0.694 mmol) were allowed to react with propargyl bromide (1.2 eq.) in the presence of K_2_CO_3_ base (3.5 eq.) and 3 ml dry acetone as solvent to synthesize 1-(prop-2-ynyloxy)naphthalene **6a,** (71%)**.** Naphthalene-2-ol (0.694 mmol) was first stirred in the presence of K_2_CO_3_ (3.5 eq.) and acetone at room temperature for 2 hour then adding propargyl bromide (1.3 eq.) to the reaction mixture and stirring for 16 hour resulted in 73% yield of 2-(prop-2-ynyloxy)naphthalene **6b**. The reaction completion was monitored by TLC. No product was formed while refluxing the reaction mixture with 1 eq. of K_2_CO_3_ base. It was surprisingly noted that increasing the eq. of K_2_CO_3_ base led to reaction completion (Fig. 3, Table 3).

**Table 3:** Optimization of reaction conditions for compounds **6a–b**.

Entry	Reactant	Product	Base	Reaction	Solvent	Time	Yield
**1**	C_10_H_8_O	C_13_H_10_O **6a**	K_2_CO_3_	Complete	Acetone	15 h	71%
**2**	C_10_H_8_O	C_13_H_10_O **6b**	K_2_CO_3_	Complete	Acetone	30 h	73%

**Figure 4:**
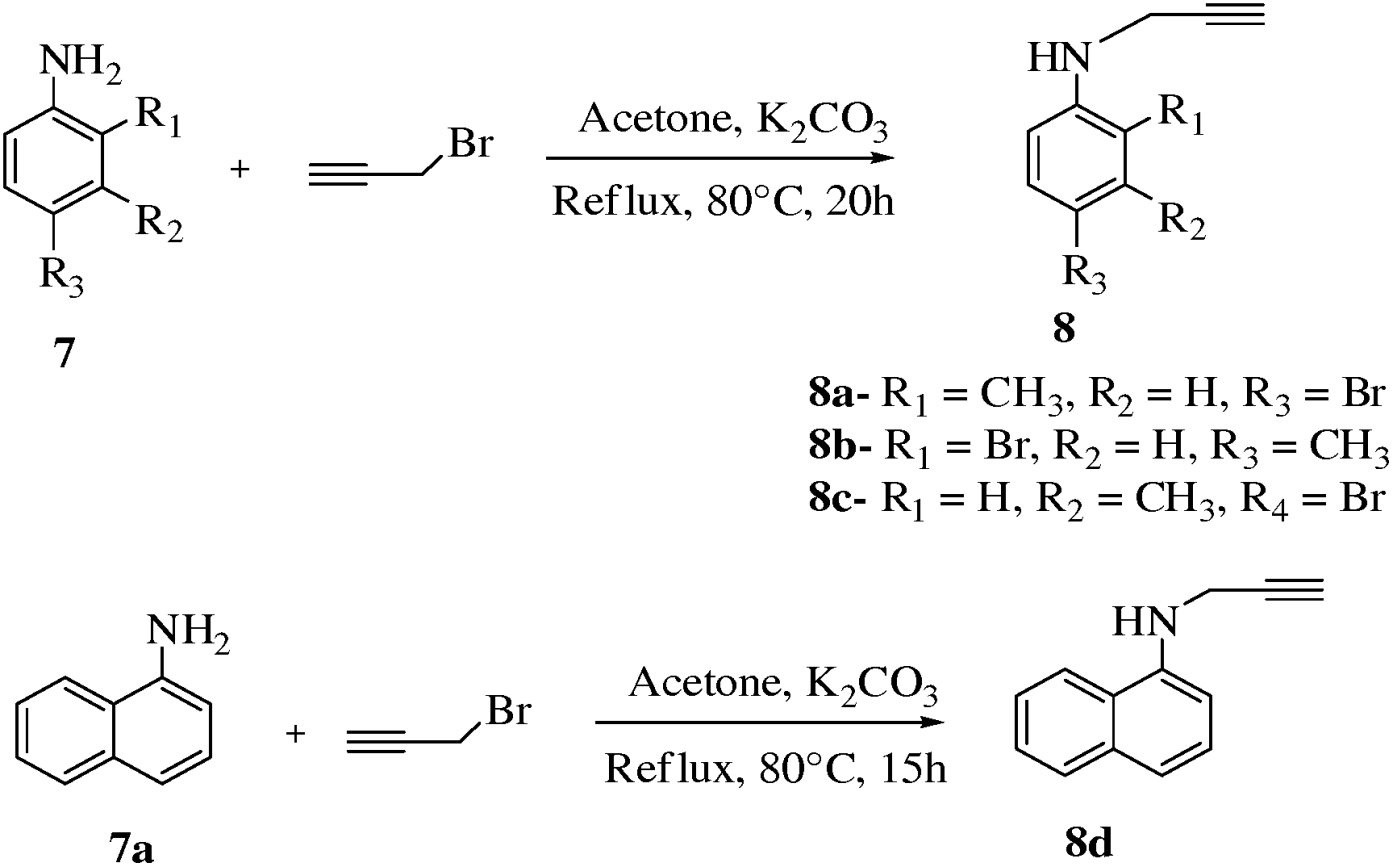
Synthesis of compounds 8a–d.

Moreover substituted aniline **7** (2.68 mmol) and **7a** (0.699 mmol) were reacted with propargyl bromide (1.3 eq.) to synthesize variously substiuted (prop-2-ynyl)anilines, **8a–8d** (Fig. 4, Table 4). It was noteworthy that reaction time was prolonged as compared to phenolic compounds to provide more harsh conditions in order to obtain desired products. In the presence of 1 eq. K_2_CO_3_ base **7a** was allowed to react with propargyl bromide (1.3 eq.), it was surprisingly noted that reaction was failed however using 2–3 eq. base proved helpful for the completion of reaction with very good yield of **8d** (69%).

**Table 4:** Optimization of reaction conditions for compounds **8a–d**.

Entry	Reactant	Product	Base	Temp.	Solvent	Time	Yield
**1**	C_7_H_8_NBr	C_10_H_10_NBr **8a**	K_2_CO_3_	80°C	Acetone	20 h	68%
**2**	C_7_H_8_NBr	C_10_H_10_NBr **8b**	K_2_CO_3_	80°C	Acetone	20 h	71%
**3**	C_7_H_8_NBr	C_10_H_10_NBr **8c**	K_2_CO_3_	80°C	Acetone	20 h	70%
**4**	C_10_H_9_N	C_13_H_11_N **8d**	K_2_CO_3_	80°C	Acetone	15 h	69%

**Figure 5:**
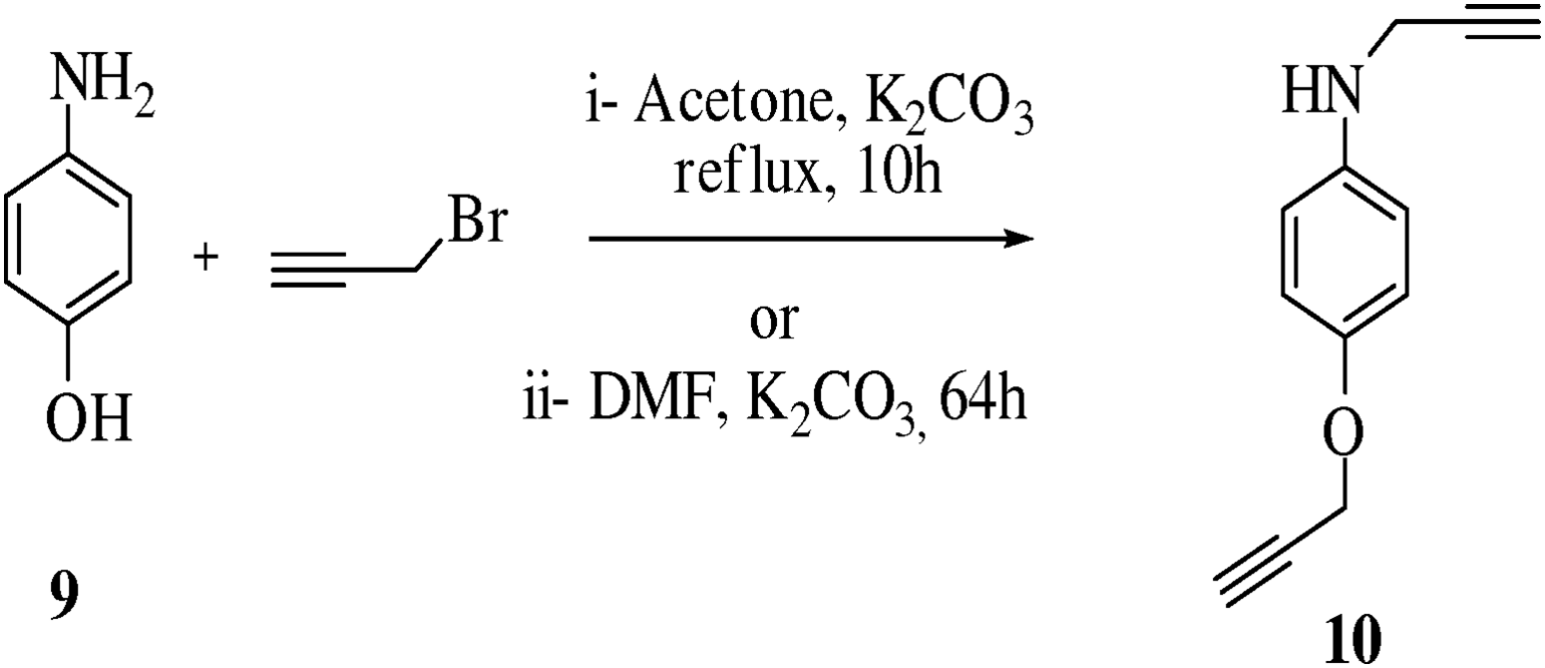
Synthesis of compound 10.

We explained a similar application of present method to obtain **10,** N-(prop-2-ynyl)-4-(prop-2-ynyloxy)aniline (Fig. 5, Table 5). It was observed that reaction was carried out at room temperature by 16 hour stirring of 4-aminophenol (0.917 mmol) and K_2_CO_3_ (2 eq.) as base with addition of propargyl bromide (2.4 eq.) in DMF as solvent during time interval of 20 min, after this reaction mixture was refluxed for 48 h under nitrogen atmosphere to obtain desired product **10**, no product was formed. It was noteworthy that when the reaction was done with K_2_CO_3_ (4 eq.) as base in dry acetone as solvent, compound **10** was obtained with 76% yield.

**Table 5:** Optimization of reaction conditions for compound **10**.

Entry	Reactant	Product	Solvents	Base	Temp	Time	Yield
**1**	C_6_H_7_ON	C_15_H_13_ON	DMF	K_2_CO_3_	80°C	64 h	0%
			Acetone	K_2_CO_3_	80°C	10 h	76%

**Figure 6:**
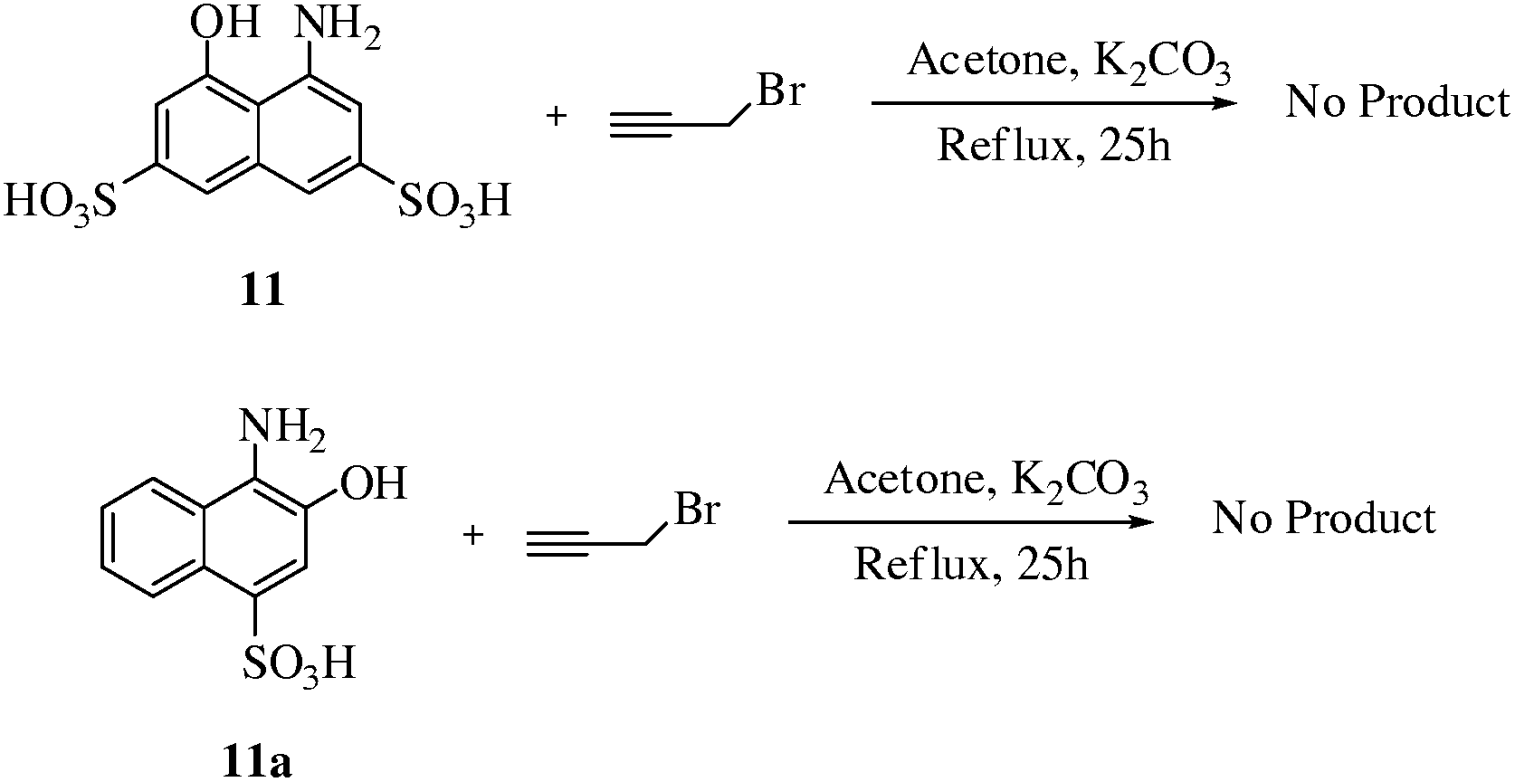
Synthesis of compounds 12a–b .

Reaction of **11** 4-amino-5-hydroxynaphthalene-2,7-disulfonic acid (0.313 mmol) and **11a** 4-amino-3-hydroxynaphthalene-1-sulfonic acid (0.418 mmol) with propargyl bromide (2.7 eq.) in basic conditions did not confirm the formation of any product. It was noted that the reaction failed even after addition of excess quantities of base and refluxing the reaction for prolonged time (Fig. 6). It might be possible that failure of reactions were due to presence of sulfonic acid functionality present in the molecule.

### Pharmacology

#### Antiurease activity

Electron donating groups enhance the activity of compounds by donating the electrons to phenyl group which enhances the binding of enzyme active site and the molecule easily [29]. Bromine containing compounds more easily inhibit the urease activity rather than compounds containing fluorine and chlorine due to lesser electronegativity values of bromine. Methyl substituent and sulfur moiety also increases the activity of compounds [30]. The compounds were tested to inhibit the activity of urease enzyme at 50 µg/ml and 100 µg/ml concentrations and their results are given in table 3. Thiourea was used as positive control with percentage inhibition activity of 65±0.01 with IC_50_ value of 23.3 at 40 µg/ml.

The results revealed that electronic effects of aryl substitutions are playing a significant role in activity manipulation. **2a, 2b** and **2e** contain bromine substitution which enhances their activity due to lesser withdrawing effect. Chloro and flouro groups decrease the activity of compounds due to more withdrawing effect. **4a** also exhibited remarkable results due to presence of methyl moieties. **2f** surprisingly gave contrasting results. As nitro group has withdrawing electronic effect, which decreases the inhibitory effect of synthesized compound. But results showed comparatively good results for the respective compound.

It was observed that all synthesized compounds **2a-10** showed moderate to very good urease inhibition activity. 4-bromo-2-chloro-1-(prop-2-ynyloxy)benzene **2a** showed comparatively good results with a percentage inhibition of 42.5±0.7 at 50 and 82.00±0.09 at 100 µg/mL with IC_50_ value of 60.2. The **2b–6a** showed good inhibition activities with percentage inhibition of 81.07±0.1, 86.45±0.07, 85.89±0.07, 79.59±0.11, 65.8±0.18, 89.42±0.05, 74.9±0.13, 65.3±0.18 at 100 µg/ml with IC_50_ values ranging between 64.92, 70.1, 67.14, 62.15, 63.7, 62.3, 70.9 and 68 µg/ml respectively (Table 6). **6b** did not show any satisfactory results.

**Table 6:** Urease inhibition studies of (prop-2-ynyloxy)benzene.

Compound	Percentage inhibition at 50 ug/mL	Percentage inhibition at 100 ug/mL	IC_50_ (ug/mL)
**2a**	42.5±0.7	82.00±0.09	60.2
**2b**	31.5±0.22	81.07±0.10	64.92
**2c**	36±0.5	86.45±0.07	70.1
**2d**	30.33±0.6	85.89±0.07	67.14
**2e**	40±0.5	79.59±0.11	62.15
**2f**	44±0.43	65.8±0.18	63.7
**4a**	37±0.34	89.42±0.05	62.3
**4b**	32±0.28	74.9±0.13	70.9
**6a**	41.33±0.41	65.3±0.18	68
**6b**	32±0.38	41.9±0.313	---
**8a**	25±0.45	55.65±0.239	90.7
**8b**	27±0.45	50.83±0.265	98.2
**8c**	32±0.38	45.26±0.295	---
**8d**	43±0.42	51.5±0.26	91.7
**10**	25±0.45	56.5±0.234	89.6
	20 ug/ml	40 ug/ml	
Thiourea	47.1±0.31	65±0.01	23.3

Aniline derivatives showed comparatively lesser activities due to lack of binding site at tertiary amine site. Ni acts as cofactor for urease enzyme, which helps in chelation of ions by compounds containing amino groups, thus activating the enzyme activity but in tertiary amines, chelation do not occur easily. Compound **8a-10** (Table 6) showed low inhibition activities with inhibition values of 55.65±0.239, 50.83±0.265, 56.5±0.234, 51.5±0.26 at 100 µg/ml with IC_50_ values of 90.7, 98.2, 89.6 and 91.7 µg/ml respectively. Compound **8c** did not show any satisfactory results.

### Nitric oxide (NO) scavenging activity

Nitric oxide is a free radical causing oxidative damage to biomolecules, finally leading to many disorders like cancer [31], aging and diabetes [32]. The synthesized compounds (**2a-10**) exhibited good nitric oxide scavenging activity. Vitamin C (Ascorbic acid) was used as a control with percentage inhibition values 38.5±0.16 at 50 and 84.1±0.12 at 100 µg/mL with IC_50_ value 50.43.

Electron withdrawing groups decreases the activity while electron donating groups enhances the nitric oxide scavenging activity. Bromine containing compounds exhibited more activity as **2c** has maximum inhibitory activity due to the presence of two bromine groups attached to aromatic ring. **4a** also exhibited remarkable results due to the presence of methyl moieties having donating effects thus enhancing the activity. **2a, 2b** and **2e** have relatively lower IC_50_ values due to electron withdrawing effects of fluorine and chlorine groups. **4b** having SO_2_ groups showed lesser inhibitory activity. Aniline derivatives also showed lower inhibitory activity as compared to that of phenolic ones.

Compounds 2,4-dibromo-1-(prop-2-ynyloxy)benzene **2c** and 4,4′-(propane-2,2-diyl)bis((prop-2-ynyloxy)benzene) **4a** were found good percentage inhibitors against NO free radicals with value 39.67±0.09 at 50 µg/mL and 77.03±0.11 at 100 µg/mL with IC_50_ value 63.8 and percentage inhibition 40±0.09 at 50 µg/mL and 74.53±0.13 at 100 µg/mL with IC_50_ value 64.4 respectively. **2a, 2b** and **2e** exhibited fairly good NO percentage inhibition (76.09±0.12, 68.44±0.16 and 77.5±0.11 at 100 µg/mL) with IC_50_ values 67.72, 67.9 and 68.8 respectively (Table 7). Compounds **2d, 4b-10** were comparatively less active to scavenge NO free radicals (69.95±0.15, 59±0.15, 63.17±0.19, 65.19±0.18, 68.65±0.16, 57.98±0.21, 62.8±0.19 and 65.5±0.18 at 100 µg/mL) with IC_50_ values 89.6, 83, 76.16, 79.4, 87.5, 78 and 78.1 for each compound respectively. 1-nitro-4-(prop-2-ynyloxy)benzene **2f** and N-(prop-2-ynyl)naphthalen-1-amine **8d** did not show any significant results and aniline derivatives showed lesser activity than that of phenol.

**Table 7:** Nitric oxide scavenging activity.

Compound	Percentage scavenging activity at 50 ug/ml	Percentage scavenging activity at 100 ug/ml	IC_50_ ug/ml
**2a**	35.67±0.09	76.09±0.12	67.72
**2b**	39.67±0.09	68.44±0.16	67.9
**2c**	39.67±0.09	77.03±0.11	63.8
**2d**	31.33±0.10	69.95±0.15	74.1
**2e**	33.33±0.1	77.5±0.11	68.8
**2f**	32±0.10	47.01±0.27	---
**4a**	40±0.09	74.53±0.13	64.4
**4b**	15.33±0.12	59±0.15	89.6
**6a**	24.33±0.12	63.17±0.19	83
**6b**	33.33±0.1	65.19±0.18	76.16
**8a**	23.33±0.11	68.65±0.16	79.4
**8b**	26.33±0.11	57.98±0.21	87.5
**8c**	33.66±0.09	62.8±0.19	78
**8d**	7.67±0.13	38.38±0.32	---
**10**	30±0.10	65.5±0.18	78.1
**Vit.C control**	38.5±0.16	84.1±0.12	50.43

### Antibacterial activity

Disc diffusion method was adopted to test the antibacterial activity of synthesized compounds **2a-10** against four different bacterial strains. Ampiciline was used as positive control to check antibacterial activity of synthesized compounds. The bacterial strains employed were: *Escherichia coli, Bacillus subtillus, Shigella flexneri, Staphylococcus aureus, Pseudomonas aeroginosa* and *Salmonella typhi.* Antibacterial activity of synthesized compounds **2a–2e, 4a, 4b** and **10** showed moderate activity against *Staphylococcus aureus* while **2f, 6a** and **8a–8d** were found to be inactive (Table 8).

**Table 8:** Antibacterial activity of (prop-2-ynyloxy)benzene derivatives.

Compound	*Staphylococcus aureus*			*Bacillus subtillus*		
	40 ug/ml	100 ug/ml	IC_50_ ug/ml	50 ug/ml	100 ug/ml	IC_50_ ug/ml
**2a**	29.13±0.12	51±0.5	97.7	42.4±0.35	34.9±0. 5	NA
**2b**	31.47±0.19	56±0.07	87.7	40.37±0.35	39.3±0.36	NA
**2c**	31.47±0.2	56±0.1	87.7	39.4±0. 75	55.01±0.25	83.79
**2d**	27.67±0.35	52±0.607	95.9	41.5±0.3	5.67±0.26	79.9
**2e**	31.99±0.07	58±0.9	85.1	9.2±0. 95	7.9±0. 75	NA
**2f**	18.66±0.3	39±0.4	NA	6.2±0. 65	57.9±0.25	87.5
**4a**	29.06±0.6	50±0.2	97.7	3.6±0. 25	33.9±0.45	NA
**4b**	26.86±0.35	53±0.7	94.21	1.7±0. 15	50.9±0.34	97.6
**6a**	–1.85±0.3	10±0.9	NA	5.5±0. 92	52.5±0. 7	92.6
**6b**	21.44±0.4	51±0.3	98.3	39.06±0.05	57.8±0.26	79.2
**8a**	16.91±0.7	33±0.8	NA	30.5±0.36	53.6±0.22	92.2
**8b**	13.39±0.5	36±0.8	NA	39.4±0. 8	43.8±0.35	NA
**8c**	15.95±0.69	35±0.2	NA	38.8±0. 15	36.0±0.39	NA
**8d**	5.85±0.8	31±0.2	NA	17.5±0. 15	45.8±0. 9	NA
**10**	24.4±0.11	52±0.1	96.3	6.13±0.35	0.7±0. 95	98.5
**Ampicilline**	29.9±0.59	73±0.51	76	9.9±0.51	9. 8±0.23	81

Hence compounds **2c, 2f, 4b–8a** and **10** have batter activity against *Bacillus subtillus.* It was noted that **2d** was found potent antibacterial against *Bacillus subtillus* showing good inhibitory action. **2a, 2b, 2e, 4a,** and **8b–8d** did not show any satisfactory result aginst *Bacillus subtillus* and were found to be inactive (Table 8). All of the synthesized compounds **2a-10** were proved inactive against *Escherichia coli, Shigella flexneri, Pseudomonas aeroginosa* and *Salmonella typhi* providing no significant results.

## Materials and Methods

### Ethics statement

No specific permits were required for these field studies. No specific permissions were required for these locations/activities because they were not carried out on privately owned or protected areas. The field studies did not involve endangered or protected species.

### General

Melting points were determined using a Buchi melting point B-540 apparatus. All reagents used were purchased from Alfa Aesar, Sigma Aldrich and E. Merck Company. ^13^C-NMR and ^1 ^H-NMR spectra were recorded on Bruker ARX 400 MHz FT-NMR spectrophotometer and Bruker ARX 600 MHz FT-NMR spectrophotometer while relishing CDCl_3_ as internal reference for the recording of spectra. The chemical shift was given in δ in ppm and coupling constant in *Hz*. EI-MS spectras were recorded on a JMS-HX-110 spectrometer, with a data system. For column chromatography technique, silica gel (70–230 mesh) and silica gel (230–400 mesh) were used. The reactions were monitored on TLC, using Merk Silica gel 60PF254 cards. The compounds were visualized by UV lamp (254, 365).

### General procedure for the synthesis of (Prop-2-ynyloxy)benzene derivatives

The synthesis of (Prop-2-ynyloxy)benzene derivatives was carried out by using four methods.

### Method A

To 1 eq (0.1 g) phenol (Figs. 1–6) added (1.2–2.7 eq.) propargyl bromide and (2–4 eq.) K_2_CO_3_ in acetone (3 ml)_._ The reaction mixture was refluxed at 80°C till reaction completion based on TLC monitoring. On completion the reaction mixture was cooled up to room temperature and solvent was removed under reduced pressure. The residue was dissolved in distilled water and then extraction was done by twice addition of methylene chloride. Organic layer was separated and dried with the help of anhydrous magnesium sulphate. Solvent was removed under reduced pressure to obtain the desired product. After evaporation and filtration process the product was purified by flash chromatography. 60/40 mixture of hexane and ethyl acetate was used as eluent [5], [33]–[34].

### Method B

1 eq (0.1 g) of phenol (Fig. 1) and (1.2 eq.) of LiH was stirred in 6 ml of THF at room temperature for 30 min, added (2 eq.) propargyl bromide to the reaction mixture and stirred overnight at room temperature. Reaction completion was monitored by TLC conditions. Evaporated the solvent under reduced pressure to obtain the final product. Product was purified by flash chromatography. 60/40 mixture of hexane and ethyl acetate was used as eluent [9], [35].

### Method C

Phenol (Fig. 1 and 3) 1eq (0.1 g) and K_2_CO_3_ (3.5 eq.) was stirred at room temperature for two hours in 0.6 ml acetone. Propargyl bromide (1.3 eq.) in 1.66 ml acetone was added in 20 min and refluxed the reaction mixture for 16 hours under nitrogen atmosphere. Reaction completion was monitored by TLC. After the reaction completion solvent was removed under reduced pressure. The residue was dissolved in distilled water and organic layer was extracted using dichloromethane thrice. Additional water was removed by drying with anhydrous MgSO_4_ [11], [36].

### Method D

1 eq (0.1 g) of 4-aminophenol (Fig. 5) and (3.5 eq.) anhydrous potassium carbonate in 0.6 ml dry DMF was stirred at room temperature for about 16 hours. 2.4 eq propargyl bromide in 0.48 ml dry DMF was added slowly in about 15 min. the reaction mixture was heated at 80°C for 48 hours under nitrogen atmosphere. Reflux conditions were used for heating the reaction mixture. Reaction completion was monitored by TLC and reaction time varied according to the condition of product. After reaction completion the solvent was removed reduced pressure. Residue was dissolved in water and then organic compound was extracted by addition of dichloromethane thrice the volume of water. Excess water was removed by adding MgSO_4_. The solvent was removed under reduced pressure and the compound separated was recrystallised using methanol thrice [11], [36].

#### Synthesis of 4-bromo-2-chloro-1-(prop-2-ynyloxy)benzene (2a)

Brown solid, M.p. 29°C, 79% Yield, ^1^H-NMR (400 MHz, CDCl_3_): δ = 7.50 (d, *J* = 2.4 Hz, 1H-Ar), 7.33–7.25 (m, 1H-Ar), 6.96 (d, *J* = 8.8 Hz, 1H-Ar), 4.74 (d, *J* = 2.4 Hz, 2H-CH_2_), 2.53 (t, 1H-acetylene).^ 13^C-NMR (100 MHz, CDCl_3_+ CD_3_OD): δ = 156.5, 134, 129.6, 122.2, 75.2, 51.2; EIMS (*m/z* -ion mode): 245 [M-H^−^]; [M-CH_2_CCH fragment]^−^ = 206; [M-Br fragment]^−^ = 165.

#### Synthesis of 2-bromo-4-chloro-1-(prop-2-ynyloxy)benzene (2b)

Brown liquid. 78% Yield, ^1^H-NMR (400 MHz, CDCl_3_): δ = 7.54 (d, *J* = 2.4 Hz, 1H-Ar), 7.23–7.15 (m, 1H-Ar), 6.98 (d, *J* = 8.8 Hz, 1H-Ar), 4.77 (d, *J* = 2.0 Hz, 2H-CH_2_), 2.53 (t, 1H-acetylene). ^13^C-NMR (100 MHz, CDCl_3_+ CD_3_OD): δ = 161, 137.5, 131, 125.2, 115.5, 76.8, 72.1, 51.2; EIMS (*m/z* -ion mode): 245 [M-H^−^]; [M-CH_2_CCH fragment]^−^ = 206; [M-OCH_2_CCH fragment]^−^ = 191; [M-Br fragment]^−^ = 165.

#### Synthesis of 2,4-dibromo-1-(prop-2-ynyloxy)benzene (2c)

Brown solid. M.p. 157.95°C, 85% Yield, ^1^H-NMR (600 MHz, CDCl_3_): δ = 7.68 (d, *J* = 2.4 Hz, 1H-Ar), 7.39–7.30 (m, 1H-Ar), 6.95 (d, *J* = 9.0 Hz, 1H-Ar), 4.76 (d, *J* = 2.4 Hz, 2H-CH_2_), 2.55 (t, 1H-acetylene). ^13^C-NMR (100 MHz, CDCl_3_+ CD_3_OD): δ = 167.9, 140.7, 127.8, 116, 76.5, 74.1, 51.2; EIMS (*m/z* -ion mode): 289 [M-H^−^]; [M-CH_2_CCH and CO fragment]^−^ = 221; [M-Br fragment]^−^ = 207; [M-OCH_2_CCH and Br fragment]^−^ = 149.

#### Synthesis of 2-bromo-4-methyl-1-(prop-2-ynyloxy)benzene (2d)

Yellow oily liquid. 74% Yield, ^1^H-NMR (400 MHz, CDCl_3_): δ = 7.27 (d, *J* = 2.4 Hz, 1H-Ar), 6.97–6.88 (m, 1H-Ar), 6.67 (d, *J* = 9 Hz 1H-Ar), 4.69 (d, *J* = 2.1 Hz, 2H-CH_2_), 2.99 (t, 1H-acetylene), 1.55 (s, 3H-CH_3_).^ 13^C-NMR (100 MHz, CDCl_3_+ CD_3_OD): δ = 157, 138.9, 129, 79.3, 75.7, 51.2, 19.8; EIMS (*m/z* -ion mode): 224 [M-H^−^]; [M-CH_2_CCH fragment]^−^ = 186; [M-Br fragment]^−^ = 145; [M-CH_2_CCH and Br fragment]^−^ = 107.

#### 2-bromo-4-fluoro-1-(prop-2-ynyloxy)benzene (2e)

Brown liquid. 79% Yield, ^1^H-NMR (400 MHz, CDCl_3_): δ = 7.02–6.97 (m, 3H-Ar), 4.72 (d, *J* = 2. 4 Hz, 2H-CH_2_), 2.51 (t, 1H-acetylene). ^13^C-NMR (100 MHz, CDCl_3_+ CD_3_OD): δ = 154.8, 124.3, 120.1, 79.4, 55.6; EIMS (*m/z* -ion mode): 231 [M-H^−^]; [M-CH2CCH fragment]^−^ = 193; [M-CO and CH_2_CCH fragment]^−^ = 165.

#### Synthesis of 1-nitro-4-(prop-2-ynyloxy)benzene (2f)

Yellow crystals. M.p. 129°C, 76% Yield, ­^1^H-NMR (400 MHz, CDCl_3_): δ = 8.216 (d, *J* = 9 Hz, 1H-Ar), 7.05 (d, *J* = 9 Hz, 1H-Ar), 4.77 (d, *J* = 2.4 Hz, 2H-CH_2_), 2.56 (t, 1H-acetylene). ^13^C-NMR (100 MHz, CDCl_3_+ CD_3_OD): δ = 166.5, 142.2, 129, 77, 57.8; EIMS (*m/z* -ion mode): 177 (M-H^−^]; [M-NO_2_ fragment]^−^ = 131; [M-NO_2_ and OCH_2_CCH fragment]^−^ = 77.

#### Synthesis of 4,4′-(propane-2,2-diyl)bis((prop-2-ynyloxy)benzene) (4a)

Light brown greasy liquid. 73% Yield, ^1^H^–^NMR (600 MHz, CDCl_3_): δ = 7.164–7.147 (m, 4H-Ar), 7.103–7.083 (m, 4H-Ar), 4.66 (d, *J* = 2.4 Hz, 4H-CH_2_), 2.90 (s, 6H-CH_3_), 2.52 (t, 2H-acetylene). ^13^C-NMR (100 MHz, CDCl_3_+ CD_3_OD): δ = 156, 151, 130.9, 117, 77.5, 31.8; EIMS (*m/z* -ion mode): 304 [M-H^−^]; [M-CH_3_ fragment]^−^ = 289; [M-CH_2_CCH fragment]^−^ = 266; [M-benzene, CH3 and OCH_2_CCH fragment]^−^ = 169.

#### Synthesis of 4,4′-sulfonylbis((prop-2-ynyloxy)benzene) (4b)

White solid. M.p. 136°C, 69% Yield, ^1^H-NMR (400 MHz, CDCl_3_): δ = 7.86–7.83 (m, 4H-Ar), 7.24–7.00 (m, 4H-Ar), 4.71 (d, *J* = 2. 4 Hz, 4H-CH_2_), 2.52 (t, 2H-acetylene). ^13^C-NMR (100 MHz, CDCl_3_+ CD_3_OD): δ = 170, 140.7, 128, 119.3, 80.4, 77.5; EIMS (*m/z* -ion mode): 325 [M-H^−^]; [M-CH_2_CCH fragment]^−^ = 288; [M-2CH_2_CCH fragment]^−^ = 250; [M- CH_2_CCH and CO fragment]^−^ = 223; [M- CH_2_CCH and 2CO fragment]^−^ = 195.

#### Synthesis of 1-(prop-2-ynyloxy)naphthalene (6a)

Light brown liquid. 71% Yield, ^1^H-NMR (400 MHz, CDCl_3_): δ = 7.77–7.73 (m, 3H-Ar), 7.45–7.32 (m, 3H-Ar), 7.18–7.09 (m, 1H-Ar), 4.80 (d, *J* = 2.0 Hz, 2H-CH_2_), 2.53 (t, 1H-acetylene). ^13^C-NMR (100 MHz, CDCl_3_+ CD_3_OD): δ = 158, 135, 130.6, 126, 77.8, 63; EIMS (*m/z* -ion mode): 182 [M-H^−^]; [M-CH_2_CCH fragment]^−^ = 143; [M-CH_2_CCH and CO fragment]^−^ = 115; [M-benzene and OCH_2_CCH fragment]^−^ = 77.

#### Synthesis of 2-(prop-2-ynyloxy)naphthalene (6b)

Dark brown greasy liquid. 73% yield, ^1^H-NMR (400 MHz, CDCl_3_): δ = 7.82–6.75 (m, 7H-Ar), 4.81 (d, *J* = 2.07 Hz, 2H-CH_2_), 3.01 (t, 1H-acetylene). ^13^C-NMR (100 MHz, CDCl_3_+ CD_3_OD): δ = 160, 128.3, 126, 122.7, 116.5, 77.5, 58; EIMS (*m/z* -ion mode): 182 [M-H^−^]; [M-CH_2_CCH fragment]^−^ = 143; [M-CH_2_CCH and CO fragment]^−^ = 115; [M-benzene and OCH_2_CCH fragment]^−^ = 77.

#### Synthesis of 4-bromo-2-methyl-N,N-di(prop-2-ynyl)aniline (8a)

Dark brown liquid. 68% Yield, ^1^H-NMR (400 MHz, CDCl_3_): δ = 7.20 (d, *J* = 1.96 Hz, 1H-Ar), 7.14–7.05 (m, 1H-Ar), 6.58 (d, *J* = 9 Hz, 1H-Ar), 3.94 (d, *J* = 2.4 Hz, 2H-CH_2_), 2.71 (t, 1H-acetylene), 2.18 (s, 3H-CH_3_), 1.18 (s, 1H-NH). ^13^C-NMR (100 MHz, CDCl_3_+ CD_3_OD): δ = 154.2, 140, 138.9, 120.4, 79, 75.3, 45, 18.5; EIMS (*m/z* -ion mode): 224 [M-H^−^]; [M-CH_2_CCH fragment]^−^ = 184; [M-Br fragment]^−^ = 144; [M-CH_2_CCH and Br fragment]^−^ = 106.

#### Synthesis of 2-bromo-4-methyl-N-(prop-2-ynyl)aniline (8b)

Dark brown liquid. 71% Yield, ^1^H-NMR (400 MHz, CDCl_3_): δ = 7.26 (s, 1H-Ar), 6.98–6.91 (m, 1H-Ar), 6.64 (d, *J* = 8 Hz, 1H-Ar), 3.96–3.90 (m, 4H-CH_2_), 2.22 (t, 2H-acetylene), 1.55 (s, 3H-CH_3_). ^13^C-NMR (100 MHz, CDCl_3_+ CD_3_OD): δ = 140.5, 137.8, 129.9, 117, 80, 76.5, 46, 20.9; EIMS (*m/z* -ion mode): 224 [M-H^−^]; [M-CH_2_CCH fragment]^−^ = 184; [M-Br fragment]^−^ = 144; [M-CH_2_CCH and Br fragment]^−^ = 106.

#### Synthesis of 4-bromo-3-methyl-N-(prop-2-ynyl)aniline (8c)

Dark brown solid. M.p. 40.85°C, 70% Yield, ^1^H-NMR (400 MHz, CDCl_3_): δ = 7.17 (d, *J* = 6 Hz, 1H-Ar), 6.63–6.55 (m, 1H-Ar), 6.36 (d, *J* = 7 Hz, 1H-Ar), 4.06 (d, *J* = 2.4 Hz, 4H-CH_2_), 2.86 (t, 2H-acetylene), 2.18 (s, 3H-CH_3_). ^13^C-NMR (100 MHz, CDCl_3_+ CD_3_OD): δ = 150.6, 135.9, 130, 117.2, 80, 75, 45, 23.8; EIMS (*m/z* -ion mode): 224 [M-H^−^]; [M-CH_2_CCH fragment]^−^ = 184; [M-Br fragment]^−^ = 144; [M-CH_2_CCH and Br fragment]^−^ = 106.

#### Synthesis of N-(prop-2-ynyl)naphthalen-1-amine (8d)

Brown greasy liquid. 69% Yield, ^1^H-NMR (400 MHz, CDCl_3_): δ = 7.78–6.7 (m, 7H-Ar), 4.11 (d, *J* = 2.40 Hz, 2H-CH_2_), 2.88 (t, 1H-acetylene), 2.26 (s, 1H-NH). ^13^C-NMR (100 MHz, CDCl_3_+ CD_3_OD): δ = 149, 135, 126, 107.6, 80.8, 75.5, 31; EIMS (*m/z* -ion mode): 180 [M-H^−^]; M-CH_2_CCH fragment]^−^ = 143; [M-HCN fragment]^−^ = 115; [M-benzene and HNCH_2_CCH]^−^ = 77.

#### Synthesis of N-(prop-2-ynyl)-4-(prop-2-ynyloxy)aniline (10)

Dark brown greasy liquid. 53% Yield, ^1^H-NMR (400 MHz, CDCl_3_): δ = ): δ = 7.24–7.03 (m, 4H-Ar), 3.98 (d, *J* = 2.40 Hz, 6H-CH_2_), 2.88 (t, 3H-acetylene). ^13^C-NMR (100 MHz, CDCl_3_+ CD_3_OD): δ = 149, 145.5, 118.7, 118.6, 76, 71, 61, 45; EIMS (*m/z* -ion mode): 185 = [M-H^−^]; [M-CH_2_CCH fragment]^−^ = 146; [M-2CH_2_CCH fragment]^−^ = 108; [M-2CH_2_CCH and CO fragment]^−^ = 80.

### Antiurease Activity

Antiurease activity of the synthesized compounds was determined by using the modified phenol-hypochlorite method [37]. Varying concentrations were used to prepare the urease inhibition curve of Thiourea (a known inhibitor of Urease) in the reaction mixture. Phosphate buffer solution of pH 7 containing 1 unit of enzyme was added about 200 µl in the test tube which was followed by the addition of 230 µl of phosphate buffer and 20 µl of thiourea from a scrupulous stock solution for the particular concentration of thiourea in the reaction mixture respectively. Test sample solutions were added instead of thiourea solution in case of test samples. The reaction mixture were mixed and incubated at 25°C for 5 min. 400 µl of urea stock (20 mM) was added in each test tube. In order to allow the action of urease on urea, all the test tubes were incubated at 40°C.

Phenol hypochlorite reagent (1150 µl) was added after 10 min incubation at 40°C. Test tubes were again incubated at 56°C for 25 min. A blue coloured complex was formed in the reaction mixture. The samples were allowed to cool for 5 min and then their absorbance values were measured at 625 nm and the %age inhibition was calculated. Experiment was repeated three times to get the SD values.

### NO Scavenging Activity

Solutions of known concentration were prepared by dissolving the synthesized compounds in appropriate solvents. [38] A mixture of sample solution (20 µl), 10 mM sodium nitropruside solution (200 µl) and phosphate buffer of 7.4 pH (1780 µl) was taken in a dry test tube and the mixture was incubated at 37°C for 2 hours. 200 µl Griess reagent (0.3% Sulphanilic acid in glacial acetic acid, 0.1% Naphthyl ethylene diamine HCL (NED): Mixed equal volume of both solutions just before use) was added after the incubation period is over. After 20 min keeping the solution at room temperature, its absorbance at 528 nm was measured. Each sample was analyzed in triplicate. Positive control of any known antioxidant and control of all samples were run in parallel. Use of negative control was to calibrate the instrument. Sample blanks were also prepared in case if the samples were coloured. Correction in the absorbance values was made by subtracting the sample blank reading from the sample reading.

### Antibacterial Activity

Disc diffusion method was used for the determination of antibacterial activity. [39] synthetic organic compounds were examined to evaluate their antibacterial potential against four different strains of bacteria i.e. *Staphlococcus aureus* (Gram + ive), *Pseudomonas- aeroginosa* (Gram−ive) and *Escherichia coli* (Gram−ive), *Bacillus subtilis* (Gram + ive), *Salmonella typhi* (Gram−ive) and *Shigella flexneri* (−ive). The synthetic organic compounds were dissolved in an appropriate solvent to get the solution of known concentration (5 µg/µl or 10 µg/µl). In order to prepare discs of different potencies known volume of extract/synthetic organic compound solution was applied on the discs, air dried and used. After preparation of the sterilized nutrient agar and nutrient broth medium, the sterilized nutrient agar medium was poured under aseptic conditions into sterile glass petri dishes/plates. A known volume of bacterial growth culture can also be poured on the surface of agar medium solidified in petri dishes. After inoculation petri plates were kept at room temperature for 15–30 minutes so that the poured medium is adsorbed. All the discs, including test sample, +ive and –ve controls were placed onto the inoculated nutrient agar medium solidified in petri dishes with the help of sterile forceps and incubated the petri plates at 37°C±2°C for 16–24 hours. Zone of inhibition was measured in mm using ordinary scale. Experiment was repeated three times to get the SD values.

## Conclusions

We have reported the synthesis of (prop-2-ynyloxy)benzene derivatives with efficient yields under mild conditions. Some of the reactions were proceeded using comparatively harsh conditions. Using more than one method helped in comparison of reaction conditions and finding out the best one. Potassium carbonate is a mild base, which provides best reaction conditions. Other bases used were comparatively harsh which may also affect the aromatic ring rendring it unfavorable for reaction products. Harsh bases like LiH, NaOH deactivate the aromatic ring. Acetone favours S_N_2 type reaction providing aprotic polar conditions so acetone and potassium carbonate were proven to be best for the synthesis of desired products. The synthesized compounds were proved to be good antiurease, antioxidant and antibacterial substances. Phenolic Compounds containing electron donating groups were proved to be better urease inhibitors and good NO scavenging activity than those compounds containing electron withdrawing groups. In contrast synthesized phenolic compounds containing both electron donating and electron withdrawing functionality gave better results against bacterial strains. It also noted Aniline derivatives showed lesser activity than phenolic compounds. The results of this study revealed that these compounds may have potential inhibitory activity and can be used as antibacterial, antiurease and antioxidant molecules.
